# Geriatric study in Europe on health effects of air quality in nursing homes (GERIE study) profile: objectives, study protocol and descriptive data

**DOI:** 10.1186/2049-6958-8-71

**Published:** 2013-11-21

**Authors:** Isabella Annesi-Maesano, Dan Norback, Jan Zielinski, Alfred Bernard, Cristina Gratziou, Torben Sigsgaard, Piersante Sestini, Giovanni Viegi

**Affiliations:** 1 INSERM, U 707: EPAR, Paris, France; 2 Université Pierre et Marie Curie – Sorbonne Univiversités, UMR S 707: EPAR, Paris, France; 3 Departments of Medical Science, Occupational and Environmental Medicine, University Hospital, Uppsala University, Uppsala, Sweden; 4 2nd Department of Respiratory Medicine, National Research Institute of Tuberculosis and Lung Diseases Warsaw, Warsaw, Poland; 5 Department of Public Health, Catholic University of Louvain, Brussels, Belgium; 6 Asthma Centre Pulmonary and critical care Department, Athens University, Athens, Greece; 7 Sect of Environment Occupation & Health Department of Public Health, Aarhus University, Aarhus, Denmark; 8 Institute of Respiratory Diseases, Viale Bracci, Siena, Italy; 9 Pulmonary Environmental Epidemiology Unit, CNR Institute of Clinical Physiology, Pisa, Italy; 10 CNR Institute of Biomedicine and Molecular Immunology, Palermo, Italy

**Keywords:** Indoor, Air quality, Comfort parameters, Elderly, Indoor, Respiratory health

## Abstract

**Background:**

Indoor air pollution (IAP) constitutes a major global public health problem requiring increasing efforts in research and policymaking that may have special significance for elderly that are likely to spend most of their day indoors and appear to be particularly susceptible to adverse effects of chemical pollutants and bio-contaminants. Yet, evidence existing on the effects of IAP in elderly is scanty. The Geriatric study in Europe on health effects of air quality in nursing homes (GERIE) study aimed to assess health effects of major indoor air pollutants and thermal conditions in elderly (> 70 years) living stably in nursing homes (NH) across Europe. Respiratory effects were particularly considered as airways and lung constitute the first target of air pollutants.

**Objectives:**

We describe here the rationale and the methods of the GERIE Study.

**Methods:**

8 nursing homes were randomly selected in 7 European countries. Twenty individuals were randomly selected in each nursing home. Major indoor and outdoor air chemical pollutants (PM_10_, PM_2.5_, PM_0.1_, formaldehyde, NO_2_; O_3_, VOC, CO_2_) and bio-contaminants (moulds, allergens) were assessed objectively with standardized procedures. Major health status indicators were assessed through a standardized questionnaire, non-invasive clinical tests and blood and urine biomarkers as well as saliva for ADN.

**Results:**

The GERIE study has given the opportunity to publish two reviews on respiratory health effects of indoor and outdoor air pollution in elderly. In addition it has provided the inventory of air quality and thermal conditions in 50 nursing homes across Europe and data on respiratory health status in 600 elderly aged 82 years in mean. Major future results will include the relationships between NH environment and health in elderly.

**Conclusions:**

The main long-term purpose of the GERIE study is to improve the health of elderly who permanently reside in nursing homes or of those who are exposed to indoor air pollution because of reduced mobility.

## Rationale

The purpose of the present paper is to present the rationale, objectives, protocol and descriptive data of the EU funded Geriatric study in Europe on health effects of air quality in nursing homes (GERIE study) aiming to contribute to the knowledge on respiratory health effects of indoor air quality and comfort parameters in elderly (http://www.geriestudy.eu).

The main challenge for the future in industrialized countries is the aging of the population, particularly the growing number of very old people, and how they live in our cities (in terms of housing and social integration) or in nursing homes (quality of healthcare and management). In industrialized countries, approximately 5% of persons aged ≥ 65 years and 20% of those ≥ 85 years are nursing home (NH) residents (http://www.uscare.com). Medical care is an essential part of NH care and this is associated with an excessive use of disinfectants and cleaning products. Disinfectants, cleaning products and bio-contaminants are the major pollutants found in NHs [1]. Inadequate ventilation can increase indoor pollutant levels by not bringing in enough outdoor air to dilute emissions from indoor sources and by not carrying indoor air pollutants out of the home. High temperature and humidity levels can also increase concentrations of some pollutants. This is a crucial issue as elderly are at greater risk of the effects of air pollution because of increased exposure and vulnerability [2,3]. There is scientific evidence that due to existing cardiopulmonary co-morbidities (cardiovascular diseases, chronic bronchitis, emphysema, and asthma), elderly suffer from reduced ability to breathe, and thereafter are greatly affected by the increased impairment that can result from exposure to air pollutants. Due to their reduced physical activities, outings and commuting, elderly result also to be more exposed to air pollutants in the place where they live. Although health effects of indoor air pollutants have been documented at the level of the general population [4], few investigations have targeted the elderly.

## GERIE objectives

The general objective of the GERIE study was to fill the gap that exists in the field of the knowledge of the effects of indoor air quality in elderly by targeting permanent residents of NHs and similar places hosting stably elderly people in Europe. Elderly living permanently in NHs were targeted in the GERIE study because, due to their situation and reduced mobility, their air pollution and other environmental exposure mis-classification is reduced so that they constitute an interesting study model. In addition, in NHs air pollutants are well characterized: the most important ones being cleaning products and bio-contaminants related to infections. The main long-term purpose of the GERIE study is to identify best practices in order to improve the health of elderly. Overall, the study will bring up also information also on mechanisms underlying the response of the organism to air pollutants in elderly.

Specific objectives of the GERIE study are to: 1) inventory on a community-wide basis the current levels of air pollutants and thermal conditions to which are commonly exposed the elderly in NHs across Europe and 2) estimate their impact on health, thus contributing an effort in the understanding of health risks in elderly. The ultimate objective is to provide a framework to help to improve air quality and thermal conditions in NHs and human health of elderly by setting health-based objectives for main air pollutants present in NHs. In order to be valuable the results had to be obtained in a sample of NH and elderly across Europe as assessed through a sample size computation.

Targeted air pollutants include those that are characteristic of indoor settings and NH in particular like particulate matter (PM) from cigarette smoke, combustion and outdoor transfer, nitrogen dioxide (NO_2_) typical of combustion and traffic, ozone (O_3_) resulting from traffic, and volatile organic compounds (VOCs) due to disinfectants, cleaning products and furniture as well as bio-contaminants following infections. They encompassed also moulds, microbes and allergens and comfort parameters like temperature, humidity and carbon dioxide (CO_2_) as a proxy of ventilation. Lastly building characteristics were considered. All these factors have been related to various health outcomes [5]. In addition, potential biomarkers of exposure or effect were considered. These contained serum cytokines as markers of inflammation and serum or urinary markers of the integrity of the pulmonary barriers (SP-D, CC16 in serum) or renal function (serum Cystatin C, urinary RBP and albumin).

## GERIE protocol

The GERIE study consisted of a European multicentre cross-sectional study that collected and integrated information belonging to several different aspects of the interaction between the NH environment and health status. To this extent a semi-individual design with individual health data and ecological environmental exposure data at the NH level was adopted. As a crucial part of health assessment, NH airborne infection control policies and COPD friendliness were appraised. Information on GERIE Study protocol and methods are available at (http://www.geriestudy.eu).

### Nursing homes sample and population

Eight NHs were randomly selected in a geographical area representative of the city in each of the 7 countries participating in the GERIE study. In each NH, at least 20 individuals aged ≥70 years were randomly selected to fill in the standardized questionnaire on health and risk factors and to undergo physical examination including non-invasive clinical tests such as breath analysis and blood and urine sampling for a total of almost 1000 individuals. This was an overestimated sample size based on the fact that there would have been refusals. Indeed, a sample of almost 600 individuals was sufficient to reach the statistical power (actually a 80 percent power) necessary to investigate the relationship between elevated exposure to indoor air pollutants (based on the 3^rd^ tertile of the air pollutant distribution) and common diseases or symptoms (15%). In each centre, the lack of representativeness of the sample of NHs was checked using the information obtained in the state of art on “Nursing Homes across Europe”. This report provides an idea of the estimated elderly population resident in homes in the seven countries. In each country, the list of residents was obtained by the managers of the NHs after having filled the terms and the conditions of the respect of the privacy.

All the individuals randomly chosen that accepted to participate in the GERIE Study were included, interviewed and underwent the physical examination including the tests and specimen collection. In case of refusal at the recruitment, other individuals were randomly chosen in the same NHs in order to attain the fixed sample size. To this extent, in each NH, 50 names of elderly were randomly selected before starting the field survey. The ranking was respected in individual selection. An information plan was prepared to explain the study method to the individuals or to their relatives and receive their informed consent to be enrolled in the study. It contained an information letter and a consent form that was signed by the participants. The study protocol underwent the approval of the Ethical committee of each country as requested by the country law.

### Environmental assessments and building characteristics

The NH setting was characterized through environmental assessments of air quality and comfort parameters and building characteristics of the NH. The buildings were inspected in detail by a technician that performed also environmental measurements at the same time period as the medical investigations.

In order to characterize at best the living conditions of the elderly, environmental assessments were performed at least in the living room, i.e. the room where the elderly persons spent the majority of their time. In some NH, several rooms were considered. Comfort and chemical paramters that were measured included room temperature, relative air humidity, PM_10_, PM_2.5_, PM_0.1_, formaldehyde, nitrogen dioxide (NO_2_); ozone (O_3_), specific volatile organic compounds (VOC) and carbon dioxide (CO_2_) concentrations. In addition, dust samples were collected from floors and chairs by using vacuum cleaning in a standardised fashion and used for analysis of allergens (cat, dog, horse, house dust mites) and microbial markers. Moreover, a simplified method to collect fungal and bacterial DNA was assessed, by means of a sterile top, from the upper part of the door frame, which contains dust accumulated during many months. The Petri-dish method [5] was used to collect airborne particles, and analyzed for allergens, bacterial DNA and fungal DNA. Lastly, acoustics (reverberation time), noise level (dBA) and illumination (lux) were measured in the living rooms.

Information about daily (24 h) mean outdoor temperature, relative air humidity and wind velocity were collected from local meteorological authorities, during the measurement days and during the colder part of the year (heating season). The following section (GERIE Study methods to assess air pollutants and comfort parameters) shows the detail of the assessments. All measurements were made during normal activities and under representative conditions. Outdoor exposure measurements of chemical and microbial pollutants were also performed during the same time period and by the same methods as the indoor measurements.

### GERIE Study methods to assess air pollutants and comfort parameters

• Temperature, relative humidity, and carbon dioxide concentrations were measured during two days by an instrument with data loggers (Q-track)

• Illumination was measured at 20–30 points in each living room by a conventional lux-meter, in two different days.

• Acoustics in the living room (reverberation time) was also measured

• Noise levels were measured in the living room at three different conditions. Empty living room with windows closed and empty living room, windows opened. These measurements were both performed during lunch time. In addition, noise measurements (dBA) during the day were performed by an integrating sound meter.

• Particles PM_10_, PM_2.5_ and ultra-fine particles (PM_0.1_) were measured during two days by portable instruments with data loggers (Dust-track and P-track)

• In addition, high flow pumps (18 L/min) were used to collect PM_2.5_, PM_10_ particles on filter, for gravimetric analysis.

• Measurements of NO_2_, and ozone was performed with two samples per living, each sampled during one week, with diffusion samples from IVL (Gothenborg, Sweden).

• Benzene and other specific volatile organic compounds (VOC) were measured by passive samplers (either the ORSA diffusion sampler or the RADIELLO diffusion sampler) during the heating season with two samples per living room (one week sampling time), and by pumped sampling on charcoal tubes (0.25 L/min; 4 hours sampling time). Analysis was performed by gas chromatography–mass spectrometry (GC-MS, [6]).

• Formaldehyde was measured during one week by another diffusion sampler (RADIELLO or other) [7].

• ELISA/monoclonal antibodies were used for allergen analysis (house dust mite, cockroach, mould allergens) [8] in settled dust and airborne dust.

• Viable and total airborne moulds and bacteria were sampled on nucleopore filter, analysed by the CAMNEA Method [5]

• Bacterial DNA and fungal DNA in settled dust (and if possible in air samples collected), were determined by quantitative PCR, in co-operation with Sangtec, Stockholm, and Ancona, Uppsala. General bacterial and fungal DNA, as well as species specific DNA for some more relevant species were analyzed. Measurement of species specific and general fungal DNA-sequences, by quantitative PCR [9] were performed in settled dust and airborne dust, collected by the Petri dish method. Recent studies indicate that bacterial DNA as such can be a potent stimulator for the immune system, and that endotoxin is a marker of bacterial DNA [10]. DNA analysis of fungal sequences has been recently developed [9].

• Chemical markers (ergosterol for fungal biomass, muramic acid for bacterial peptidoglycan, and 3-hydroxy fatty acids for endotoxin) were analyzed in settled dust sampled by vacuuming floor and chairs according to a standardized protocol and analyzed by gas chromatography/ tandem mass spectrometry (GC-MSMS) [11,12].

• Microbial particles by an electrostatic samples (5 L/min flow) were used for viral analysis.

• Irritants from disinfectants (e.g. chlorine) were determined by classical analysis.

Details on constructions, materials, type of ventilation system, signs of building dampness, information on physical space e.g. m^2^/person, m^2^ of the living room/shared rooms were collected using a standardized form.

To ensure the quality and reproducibility of data collection, a specialized Environmental Team was established. Before the beginning of the field survey, members of the Environmental Team organized a technical meeting to which at least one member of each centre participated. The aim of the meeting was to describe the methods to be used and to provide hands-on training to local operators who were involved in the field survey. At least one member of the Environmental Team participated in the field survey in each centre, with the help of the local operator trained in the preliminary technical meeting. After participating in the survey with the supervision of a member of the Environmental Team, local operators could become members of the team and participate as team member in surveys in other centres.

### Health assessments

In the GERIE Study, health status was assessed through a questionnaire, interview tests, clinical investigations and biomarkers (Table 1).

**Table 1:** GERIE Study methods to assess health outcomes

**Tool**	**Compulsory or facultative**	**Health outcome**
**Questionnaire and tests**	Compulsory (questionnaire)	Respiratory symptoms and diseases
	Facultative (tests)	Depression
		Cognitive function comorbidity
**Clinical tests**		
Electrocardiogram	Facultative	Heart’s electrical activity
Spirometry,	Compulsory	Lung function
Saturometry	Compulsory	Oxygen saturation of arterial blood.
Tear film stability (BUT)	Compulsory	Functionality of the eye
Acoustic rhinometry	Compulsory	Nasal patency
CO in exhaled air	Compulsory	Effective indicator of smoking
NO in exhaled	Compulsory	Lower airways inflammation

*Questionnaire and tests:* An epidemiological questionnaire composed of several sections was administrated by trained personnel to all the selected elderly individuals. For the purpose of the survey and because elderly people get tired rapidly, only the respiratory health and comorbidity section were considered as compulsory. Facultative sections of the questionnaires and tests included a depression scale and test interviews in order to identify the presence of depression and cognitive problems to evaluate cognitive performance and quality of life that were proposed to individuals that agree. Respiratory symptoms and diseases were assessed using a standardized questionnaire, which was derived from the European Respiratory Health Survey (ECRHS) [13] and the Three-City Study, which has been conducted on cognitive decline and vascular aging in older French adults [14,15]. This questionnaire also contained questions on personal factors and medical background data, such as smoking habits, alcohol consumption, medication consumption, socio-economic conditions, familial status, occupational history, exposures in the home environment in the past as well as brief information about other morbidities. Risk factors for illness and disability were recorded. As previously said, questions and tests on cardiovascular diseases, depression and dementia cognitive skills and others were proposed as facultative.

*Clinical investigation:* All clinical investigations were performed in the NH, in the same period as the environmental survey, by an experienced nurse or specialist physician, under the supervision of at least one member of a specialized Clinical Team formed according to the same rules as described for the Environmental Team above. A technical meeting to prepare and train local operators involved in the field survey was held at the same time as the Environmental technical meeting. The clinical investigation included measurement of heart’s electrical activity through electrocardiogram, lung function with spirometry, oxygen saturation of arterial blood with saturometry, functionality of the eye with tear film stability (BUT), nasal patency measured by acoustic rhinometry, CO in exhaled air for detecting smoking. Furthermore, NO in exhaled air was performed in order to assess upper airways inflammation. All the assessments were performed using the same portable devices in all the centres and according to a standardized protocol (Dr Isabella Annesi-Maesano, Paris (spirometry and electrocardiogram), Prof. Dan Norback, Uppsala (BUT), and Prof Torben Sisgaard, Aarrhus (acoustic rhinometry, NO, CO, breath condensate) provided the device). Field personnel were trained for the purpose of GERIE. Spirometry was performed according to the guidelines of the European Respiratory Society. Oxygen saturation of arterial blood was measured through a pulse oximeter device attached to the earlobe or fingertip. Electrocardiogram (including blood pressure assessment) was performed according to the guidelines of the European Society of Cardiology. Tear film stability was estimated in each subject by two standardized methods: by measuring the time the subject could keep the eyes open when watching a fixed point at the wall [16] and directly using a small eye microscope (Keeler Tearscope Plus. Keeler UK). Acoustic rhinometry (Rhin 2000; wideband noise; continuously transmitted) was performed under standardized forms (sitting), after 5 minutes of rest, and prior to the nasal lavage [17]. Carbon monoxide (CO) was measured in exhaled air using a portable device (Smokerlyzer, http://www.bedfont.com). Lastly NO was measured in exhaled breath by a portable equipment (NIOX MINO) according to ERS guidelines.

*Biomarkers:* After approval by the institutional ethics committee, one blood sample (10 ml) and one spot urine sample were collected for each elderly in order to assess various biomarkers as well as saliva for DNA. Biomarkers included:

• Serum cytokines

• Sera were assayed for an array of inflammatory cytokines by means of a multiplex cytofluorimetric assay (Luminex). The Luminex technique enables the measurement of many different analytes in the same test with a minimum of test material. (100 μl). In each individual, we included markers for acute inflammatory responses (TNF-alpha and IL1, IL-4, IL-6, IL-8). All these analyses were performed in a centralized laboratory in Arrhus (Prof. Torben Sisgaard, Aarrhus).

• Serum or urinary markers reflecting the integrity of the pulmonary barriers (SP-D, CC16 in serum) or renal function (serum Cystatin C, urinary RBP and albumin) according to established methods [18].

In serum, we measured Clara cell protein (CC16) and surfactant-associated protein D. These two proteins have been validated as markers of the integrity (permeability or integrity of cells secreting them) of the lung epithelium. Not only these markers have been found to be altered in a variety of lung disorders (COPD, fibrosis…) and of lung toxicants, but recent studies suggest they might be predictive of an increased risk of mortality. We also measured serum Cystatin C, a sensitive and reliable marker of the glomerular filtration rate that has been found to predict mortality in elderly. In urine, we determined albumin and retinol-binding protein (RBP) to assess the integrity of the glomerular filter and of the proximal tubule respectively. These renal markers are frequently altered in elderly as a result of degenerative (diabetes) or cardiovascular diseases. Concentrations of urinary proteins were adjusted for creatinine. All these markers were determined using sensitive immunoassays in a unique laboratory in Brussels (Prof. Alfred Bernard).

### Airborne infection control policies and “COPD friendliness”

One *ad hoc* work-package included: 1) a global questionnaire survey to be conducted in all the NHs as well as an additional questionnaire for “COPD friendliness” derived from the “asthma-friendly school checklist” (http://www.lungusa.org) and 2) a satellite study to be conducted only in one centre with objective assessment of infections.

1) All nursing homes

A questionnaire survey was conducted in people living or working in all the European NHs recruited for the environmental studies (NH residents, caregivers, and administration staff). The questionnaire contained information on a variety of aspects including the presence and characteristics of Infection Control and/or Indoor Air Quality policy, incontinence management practices; immunization policy about residents and health caregivers; occurrence and type of infection outbreaks (particularly respiratory, urinary, cutaneous and gastrointestinal infections); presence of infection control guidelines for indwelling devices, antimicrobial resistant pathogens, diarrhoea, and pressure ulcers; employee education regarding infection control, smoking, asthma and COPD, tobacco smoking policy, availability of tobacco smoking programs for NH residents and caregivers.

The questionnaire was derived from published experiences and was validated by site inspection and by analysis of clinical records in a randomized sample [19].

2) Satellite study

To provide a more thorough assessment of the possible impact of airborne infection control measures, a satellite study was conducted in one of the selected centres (Prof. Piersante Sestini, Siena) where serum samples were specifically obtained from each NH resident to evaluate seroepidemiology of influenza virus A and B using enzyme-linked immunosorbent assay. In addition, colonization by *pneumococci* was evaluated in residents and caregivers using throat swab cultures and PCR analysis using standardised methods. Infection outbreaks by influenza, respiratory syncytial virus (RSV) and metapneumovirus were actively monitored by PCR analysis of throat swabs of suspected cases. Results should be used to modify the infection control policy by addressing all the critical factors detected during the local assessment. Depending on the findings of the preliminary survey, these could include changes in air quality control (i.e. Improved ventilation), vaccination (i.e. Pneumococcal vaccine), caregiver vaccination and control (including antiviral prophylaxis when a case occurs) during influenza season, more accurate monitoring, and other *ad hoc* measures, according to the “Infection Control Measures for Preventing and Controlling Influenza Transmission in Long-Term Care Facilities” of the Centre of Disease Control (http://www.cdc.gov/flu/professionals/infectioncontrol/longtermcare.htm).

Both study protocols underwent the approval of the Ethical committee in Siena according to the Italian law. The elderly were informed. A written consent was signed.

## Epidemiological and statistical analysis

Classical methods of epidemiology and statistics were used to analyse GERIE data. Health outcomes were defined according to the existing literature. They consisted of the prevalence of respiratory diseases or symptoms estimated according to age (in class), sex and centre and of results of clinical investigations and biomarkers that were mostly continuous variables. For each indoor air pollutants, the mean concentrations during the measurement period were provided in μg/m^3^. Similarly, for comfort parameters the mean was computed. The frequency of building characteristics was expressed as percentages. Exposure to indoor air pollutants and comfort parameters of the individuals participating in each survey was defined according to the median of the distribution in most analyses. However, it was not excluded to correlate air pollution concentrations taken as continuous variables with health outcomes measured in continuous (HTA, Forced Expiratory Volume in 1 sec…). When appropriate, the non-independence of data due to the fact that elderly from the same NH shared the same environment was taken into account through generalized estimating equation (GEE) models that are used to estimate the parameters of a generalized linear model with a possible unknown correlation between outcomes. In addition, multi-level models were used to consider the different levels of the investigation, namely individual, NH, and centre respectively. Uni- and multi-pollutant models were developed.

Potential confounders of the relationships between exposure and health indicators were defined as usually and taken into account in the analyses. Expected confounders include sex, social class, education centre, active and passive smoking, previous exposure to air pollutants, co-morbidities… Past exposures to occupational and environmental hazards were assessed also retrospectively. Exposure to outdoor pollution was modelled using appropriate approaches (proximity models of dispersion of air pollutants).

In the long-term, data collected in the GERIE study will be employed in the search for gene*environmental interactions in the response to indoor pollutants and in the expression of the disease.

## Descriptive data

The total number of the elderly living permanently in the NHs selected in each centre and that participated in the GERIE study was 600, corresponding to a participation rate of 50% among the eligible persons. The elderly sample size was consistent with the computed sample size. The survey lasted 2 years. Data were collected between February and March 2009 in Sweden (Uppsala), June 2009 in Poland (Warsaw), December 2009 in Greece (Athens), between May and July 2010 in France (Reims), February 2011 in Italy (Arezzo), September 2011 in Denmark (Aarhus) and in October 2011 in Belgium (Brussels). The participation to the GERIE protocol is described in Table 2.

**Table 2:** Participation to the clinical exam (questionnaire and clinical tests) by country in the GERIE Study

**Country**	**N**	**Date of visit**	**Core Q**	**Optional Q sections and tests**	**Nurse Q**	**NH Q**	**Spiro-metry**	**eCO**	**eNO**	**Saturo-metry**	**Rhino-metry**	**Tear film**	**Saliva**	**Urine**	**Blood**
**Sweden**	55	23 February - 8 March 2009	☑	X	X	X	☑	☑	☑	☑	O	☑	X	☑	X
**Poland**	170	15 - 23 June 2009	☑	X	☑	☑	☑	☑	☑	☑	O	X	☑	☑	☑
**Greece**	83	December 2009	☑	☑	☑	☑	☑	☑	☑	☑	X	X	☑	☑	☑
**Italy**	63	February 2011	☑	X	O	O	☑	☑	☑	☑	X	☑	☑	☑	☑
**France**	134	May-July 2010	☑	X	☑	☑	☑	☑	☑	☑	X	X	☑	☑	☑
**Belgium**	35	June 2011	☑	X	X	X	☑	X	X	X	X	X	X	☑	☑
**Denmark**	59	October 2011	☑	X	X	X	☑	X	X	X	X	X	X	☑	☑

☑ = Available data.

O = Data that have not been analyzed.

X = Test or questionnaire not performed.

The objective of targeting elderly was obtained. The GERIE population was aged on average 82 years. 66% of them were aged over 80 years and 74% were women. Mean BMI was 26.8 kg/m^2^. Regarding education, 55 (11%) had never been to school and 173 (34%) attended only primary studies. The highest school level attained was in Poland with 25 (16%) of the elderly in a four year college. 210 (40%) of participants declared that they smoked. Regarding respiratory health status, breathlessness was the most frequently respiratory outcome reported with a prevalence of 46%, the highest prevalence was in Poland with 62% of the participants. 7% of them had asthma and COPD, 29% cough, 24% phlegm and 14% past year wheezing.

Indoor mean temperature in the 50 NHs of the GERIE study was 23°C; the least value was measured in Greece with 22°C. Mean relative humidity was on average 37%, the highest value was in Brussels 57%. Mean concentration of CO_2_ was less than 1000 ppm in all NHs; the mean being 572 ppm. Only 105 (19%) individuals had an adequate ventilating system in the NHs in which they reside. The mean concentrations of PM_10_, PM_0.1_, formaldehyde, NO_2_ and O_3_ measured in all countries were respectively: 29.8 μg/m^3^, 12907 pt/cm^3^, 7.21 μg/m^3^, 20.1 μg/m^3^ and 21.1 μg/m^3^. The highest values of PM_10_, PM_0.1_ and NO_2_ were observed in Athens with 56 μg/m^3^, 23839 pt/cm^3^ and 36.2 μg/m^3^, respectively. For formaldehyde it was in Aarhus with 13.7 μg/m^3^ and for O_3_ in Brussels with 42 μg/m^3^. Total fungal and *Aspergillus/Penicillium* DNA was detected in most samples, both by pumped filter sampling and by the Petri dish sampling. *Aspergillus versicolor* DNA was detected in 14% of the filter samples and 28% of the Petri dish samples. *Stachybotrys Chartarum* DNA and *Streptomyces* DNA were uncommon, and could only be detected in the Petri dish samples. There were large variations of fungal DNA-levels between the rooms in the NHs. There was no standard values for acceptable levels of fungal DNA in indoor environments. Outdoor levels of fungal DNA was measured by pumped sampling outside 7 NHs (3 samples in Uppsala, one in Warsaw, one in Arezzo, and one in Reims). The mean indoor/outdoor ratio was 1.24 for total fungal DNA and 1.18 for *Aspergillus/Penicillium* DNA, but there were large variation in the indoor/outdoor ratio as well as the outdoor levels. The highest outdoor levels were found in Uppsala and Warsaw, and the much higher indoor levels found by the pumped filter sampling in these two centres could be explained by high outdoor levels. There was no correlation between fungal DNA levels measured by pumped air sampling and Petri dish sampling. This could be due to different sampling periods (1 day versus 1 week) as well as different sampling properties for the two methods. The filter method can sample smaller particles (sub-micron) but the Petri dish method will sample larger particles by sedimentation. There were variations in mean levels of indoor and outdoor air pollution and bio-contaminants amongst centres.

## GERIE publications

So far, the GERIE Study (Table 3) has engendered 3 publications: 2 in the frame of a state of art of existing data on the effects of air pollution on health in elderly and 1 using a biomarker.

1) A systematic review on health effects of outdoor air pollution that has been published in the IJTLD in 2012 [20]. Compared to the rest of the population, the elderly are potentially highly susceptible to the effects of outdoor air pollution due to normal and pathological ageing. The purpose of the present review was to gather data on the effects on respiratory health of outdoor air pollution in the elderly, on whom data are scarce. These show statistically significant short-term and chronic adverse effects of various outdoor air pollutants on cardiopulmonary morbidity and mortality in the elderly. When exposed to air pollution, the elderly experience more hospital admissions for asthma and chronic obstructive pulmonary disease (COPD) and higher COPD mortality than others. Previous studies also indicate that research on the health effects of air pollution in the elderly has been affected by methodological problems in terms of exposure and health effect assessments. Few pollutants have been considered, and exposure assessment has been based mostly on background air pollution and more rarely on objective measurements and modelling. Significant progress needs to be made through the development of ‘hybrid’ models utilizing the strengths of information on exposure in various environments to several air pollutants, coupled with daily activity exposure patterns. Investigations of chronic effects of air pollution and of multi-pollutant mixtures are needed to better understand the role of air pollution in the elderly. Lastly, smoking, occupation, comorbidities, treatment and the neighbourhood context should be considered as confounders or modifiers of such a role. In this context, the underlying biological, physiological and toxicological mechanisms need to be explored to better understand the phenomenon through a multidisciplinary approach

2) A systematic review on health effects of indoor air pollution published in J. Environ. Sci. Health, Part A in 2013 [21]. The purpose of this review was to summarize current knowledge on adverse respiratory effects of indoor air pollution in individuals aged over 65 years, by presenting existing epidemiological evidence. Data on respiratory effects of indoor air pollution in elderly were scanty. Most of them showed significant relationships between exposure to various indoor air pollutants and various short-term and long-term respiratory health outcomes such as wheezing, breathlessness, cough, phlegm, asthma, COPD, lung cancer and more rarely lung function decline. The most consistent relationship is found between chronic obstructive pulmonary disease (COPD) and environmental tobacco smoke (ETS). Data for other air pollutants are sparse. Further studies in the elderly population are needed in order to define causal relationships between exposures to indoor air pollution and underlying mechanisms in this sub-population.

3) A significant link between urinary cadmium and age and urinary proteins [22]. At low Cd exposure levels, U-Cd and age are associated through nonlinear and nonmonotonic relationships that appear to be driven mainly by recent Cd intake and physiological variations in the excretion of creatinine and proteins. Cadmium is a marker of exposure to passive smoking and in addition due to its toxicity is related to various health effects.

**Table 3:** GERIE Study Consortium

	
**France**	Epidemiology of Allergic and Respiratory Diseases Department, UMR-S U707 INSERM and UPMC-Paris6, Medical School Saint-Antoine, Paris
	- **Dr Isabella Annesi-Maesano**,
	*Project Co-ordinator, in charge of the overall project management in cooperation with University “Pierre et Marie Curie” (UPMC).*
	- **Dr Jean-François Bertholon**
	- **Dr Malek Bentayeb**
	- **Soutrik Banerjee**
	- **Cécile Billionnet**
	- **Franck Vibert**
	Service de Pneumologie, CHU Reims, Reims
	- **Dr François Lavaud**
**Belgium**	Unit of Toxicology, Catholic University of Louvain, Louvain
	- **Prof Alfred Bernard**
	- **Xavier Dumont**
**Denmark**	Dept Environmental & Occupational Med Institute of Public Health University of Aarhus, Aarhus
	- **Prof Torben Sigsgaard**
	- **Gitte Juel Holst**
**Greece**	Asthma Center Pulmonary and Critical Care Department, Athens University. Greece
	- **Prof Gratziou Cristina-Georgia**
	- **Dr Kostas Eleftheriou**
**Italy**	Servizio di Pneumologia, Università degli Studi di Siena, Siena
	- **Dr Piersante Sestini**
	Unità di Epidemiologia Ambientale Polmonare (Pulmonary Environmental Epidemiology Unit), Istituto di Fisiologia Clinica CNR (CNR Institute of Clinical Physiology), Pisa
	- **Prof Giovanni Viegi**
	- **Sonia Cerrai**
	- **Giuseppe Sarno**
**Sweden**	Department of Medical Sciences/Occupational and Environmental Medicine, Uppsala University, University Hospital
	- **Prof Dan Norback**
	- **Prof Gunilla Wieslander**
**Poland**	National Research Institute of Tuberculosis and Lung DiseasesPlocka 26, Warsaw
	- **Prof Jan Zielinski**
	- **Dr Jacek Nasilowski**

Forthcoming results from the GERIE include:

1) the knowledge of potential health hazards to which elderly people are exposed in NHs, due to exposure to thermal conditions and to the presence of air pollutants;

2) standardized data on NH across Europe;

3) in the long term whenever possible, the provision of data of utility in the implementation of indoor air quality guidelines in NHs.

## Discussion

Although an important goal is that each generation have better health among its older adults than preceding generations, age-related increases in the prevalence of chronic diseases and injuries or their *sequelae* are not expected to disappear. Older adults will continue to experience more chronic conditions than younger persons, experience more activity limitations and disability related to chronic disease, use more health-care resources because of chronic diseases, and have multiple chronic conditions (comorbidities) among the oldest of the elderly. It has been suggested that air pollution could precipitate health status in elderly people because of their frailty.

Thereafter the justification for conducting GERIE is both societal and scientific.

• Europe’s population is getting older (the greying for Europe).

• By 2030, the EU will have 34.7 million citizens aged over 80 (compared to 18.8 million today) and the baby-boomer generation become senior citizens (http://ec.europa.eu/employment_social/news/2005/mar/demog_gp_en.html). The impact on the whole of society of such demographic changes in terms of health and economic costs could be reduced by improving health status and quality of life of the elderly. One way to obtain it is through the prevention of the exposure to the contaminants that in elderly people are responsible for severe morbidity as well as for mortality. This passes through the assessment of exposure and of the burden associated to prolonged exposure to contaminants in elderly populations.

• The knowledge on adverse effects of air pollution in elderly is scanty.

Sparse population data indicate an ongoing threat to the health of the elderly population as the elderly appear to be particularly susceptible to air pollution [1-5,13-18,20,21]. However, the extent of the impact of air pollution in elderly is not exhaustively assessed. Similarly, the basis of the increased sensitivity in elderly is not known but it is likely that it is linked to age-related impaired function of the lung and deteriorated health status. These phenomena are influenced by a number of factors, both individual and environmental. Furthermore, elderly people spend most of their time indoors where the concentrations of several pollutants can be many times higher than outdoors and they are therefore at increased risk of being exposed to indoor air pollutants compared to the rest of the population [23]. Although at low concentrations, indoor air pollutants present in NHs may have important biological impact on the health of elderly living there permanently because of the long exposure periods. Also, NHs present extremely high concentrations of disinfectants, cleaning products and bio-contaminants, such viruses and bacteria, due to the conditions of confinement found there. As a consequence, elderly living permanently in NH constitutes a valid model for the study of the effects of air pollutants. Furthermore, there are still few studies having related biomarkers to indoor air pollution [24,25]. A variety of mediators are involved in the host response to air particles, and several cytokines and inflammatory mediators have been proposed as markers of mucosal damage and inflammation, including TNF, GM-CSF interleukin 1, 4, 6, 8, cationic protein, nitric oxide and other substances. Informative samples were obtained non-invasively using exhaled NO. New techniques for the multiplex analysis of several cytokines on the same sample have been recently developed and have been used for analysis of breath condensate in patients with respiratory diseases, but they have not used yet to characterize the response to pollutants in the serum. Among clinical measurements, tear film stability and acoustic rhinometry are simple non-invasive methods which may be used in children to evaluate respectively ocular and nasal irritation. To our knowledge, the GERIE study is the first to investigate these relationships in elderly persons. GERIE is the first study to use different sources of information in relation to adverse health reactions of environmental hazards in older adults. It will contribute to a better knowledge of the role of biomarkers in the assessment of the role of atmospheric pollution.

Lastly, the GERIE study will deal with the multi-pollutant issue. At present, at the EU level there is not a general recommended approach to conduct the risk assessment for chemical mixtures or for combined effects due to concomitant exposure to different chemicals through different routes. Due to the complexity of indoor air pollution and its variability with time, estimation of risk associated with exposure to the complex mixture as such and the generalization of the obtained results is debated. The GERIE study will focus this need. The chemical mixtures or combined effects will be characterized using statistical methods and modelling that have been developed in this respect [26].

## Competing interests

The authors declare that they have no competing interests.

## Authors’ contributions

IAM, DN, JZ, AB, CG, TS, PS, and GV: Equal contributors of the manuscript. IAM: Study design, Project Coordinator, Significant contributor to written manuscript. All authors read and approved the final manuscript.
